# Effect of counselling during pulmonary rehabilitation on self-determined motivation towards physical activity in people with chronic obstructive pulmonary disease – protocol of a mixed methods study

**DOI:** 10.1186/s12890-017-0457-8

**Published:** 2017-08-17

**Authors:** Anne-Kathrin Rausch-Osthoff, Nicola Greco, Ariane Schwank, Swantje Beyer, David Gisi, Mandy Scheermesser, André Meichtry, Noriane Sievi, Thomas Hess, Markus Wirz

**Affiliations:** 1 Zurich University of Applied Sciences, Institute for Physiotherapy, Technikumstrasse 71, 8401 Winterthur, Switzerland; 2 Kantonsspital Winterthur, Institute for Physiotherapy, Brauerstrasse 15, 8401 Winterthur, Switzerland; 3 Kantonsspital Winterthur, Pneumology, Brauerstrasse 15, 8401 Winterthur, Switzerland; 4 Zurich University Hospital, Pneumology, Rämistrasse 100, 8091 Zürich, Switzerland

**Keywords:** Behaviour change, Physical activity promotion, COPD, Motivational interviewing

## Abstract

**Background:**

Physical activity promotion in people with Chronic Obstructive Pulmonary Disease (COPD) is focus of research and public health. Patient-centred interventions like counselling are promising approaches to help patients reducing sedentary behaviour. Aim of the present study is to investigate if a physical activity counselling program during pulmonary rehabilitation increases physical activity level in daily life in people with COPD.

**Methods:**

A two-armed, single blind randomised controlled trial including 56 people with COPD will be conducted in an outpatient pulmonary rehabilitation. Patients will participate in a 12-week-rehabilitation program; individuals randomized to the interventional group will additionally participate in five counselling sessions with a physiotherapist, based on the principles of motivational interviewing. The participants’ physical activity level will be measured using an accelerometer (*SenseWear Pro®*) before, directly and 3 months after pulmonary rehabilitation. Semi-structured interviews will be conducted to learn more about barriers and facilitators regarding daily physical activity.

**Discussion:**

If the strategy successfully improves the physical activity level in people with COPD, counselling might be implemented in pulmonary rehabilitation.

**Trial registration:**

Clinical Trials.gov NCT02455206 (05/21/2015), Swiss National Trails Portal SNCTP000001426 (05/21/2015).

## Background

Physical inactivity in daily life is a prominent feature in people with Chronic Obstructive Pulmonary Disease (COPD) [1–5]. The amount of daily physical activity (PA) gradually declines with the severity of COPD [5–7]. In addition, PA is associated with the number of hospitalizations [8–10] and recognised as the strongest predictor of all-cause mortality [9, 11] in these patients. A downward circle of dyspnoea induced by PA may lead to a shift in patients’ lifestyle resulting in a vicious circle of decreased exercise tolerance, which in turn further reduces activity levels and increases social isolation and depression [12]. In addition, results from the PERCEIVE study indicated that patients’ perception of their COPD impacted the activities of daily living [13].

Physical inactivity became topic of great interest since both the American Thoracic Society (ATS) and the European Respiratory Society (ERS) stressed the fact that long-term self-management and adherence to exercise at home should be the primary goals of pulmonary rehabilitation programs (PR) [14]. Research has demonstrated sufficiently that PR is beneficial to people with COPD as it improves exercise capacity and locomotor muscle strength [15, 16]. Although the main purpose of PR should be enhancing PA, the impact of PR on PA in people with COPD is still unclear [17, 18]. Mantoani and colleagues [19] showed in a literature review that the intervention of 13 studies demonstrated a positive effect while the strategy of seven studies had no effect. Mantoani and colleagues stressed the fact that objectively assessed improvements were only shown by studies with an intervention phase equal or longer than 12 weeks duration, even though the increments in activity level ranged between 10 and 20%.

Apparently there is an eloquent difference between functional capacity status and daily PA, as the latter may be elicited by the habitual and environmental factors of sedentary patients. However, in order to be meaningful for people with COPD, PR-induced improvements need to be translated into changes in PA and participation in everyday situations. An alternative strategy to improve physical activity levels may be a lifestyle PA counselling program to help the patients to translate PR-induced improvements in exercise capacity during daily living. It may be postulated that implementation of comprehensive counselling program during PR is the strategy of choice [20]. One promising approach is counselling based on motivational interviewing (MI). According to Miller and Rollnik [21] this conversation method is client-centred and directive. It focusses on the exploration and evocation of a person’s intrinsic motivation to change his/her status quo regarding daily physical activity. Hereby MI emphasises the attitude of rather seeing a person ambivalent than unmotivated in regards to behavioural change. Consequently, it is the therapist’s intension to detect a patient’s ambivalence and to further resolve it. This attitude is called “MI spirit” and is based on four principles such as; be empathetic, develop discrepancy, roll with resistance and support self-efficacy [22]. The process to change the problem behaviour can be divided in two phases; first to create motivation to change and second to commit to change. The MI method is self-defined and not tailored. Several authors investigated the effects of PA counselling based on MI added to PR in people with COPD [23–25]. De Blok and colleagues [23] compared the short-term effects of a lifestyle PA counselling program with feedback of a pedometer during a nine-weeks PR on daily step count to PR alone. Twenty-one individuals with moderate to severe COPD were randomized into two groups. Four counselling sessions a 30 min were performed by physiotherapists following the principles of MI. The experimental group increased the mean daily step count by 69% for continuous 6 days without PR, whereas the control group increased by 19% compared to baseline. Effects were not statistically significant, however the between-group effect size was d > 0.8. This was the first study using a feedback-tool to improve motivation which gave useful information for following trials evaluating long-term effects in a larger patient group living in the region of Groningen [26, 27]. However, PA was quantified by using a pedometer, but objectively measuring PA by using multiaxial accelerometer seems to be the most accurate field based estimate of PA [28, 29]. Burtin and colleagues [24, 25] investigated as well the additional effect of counselling during PR in PA levels in 80 people with moderate to very severe COPD. To measure PA levels objectively an accelerometer was used before and after 3 months and after 6 months of PR. Those individuals randomized to the intervention group participated in eight individual counselling sessions a 20 to 30 min with a physiotherapist based on principles of MI. Controls received a sham attention program. Unfortunately, the study failed to show any improvements regarding PA after 6 months. The authors reflect it could be more effective to put the counselling sessions at the end respectively short after the end of PR. Furthermore, the counselling providers had no prior experience with MI. They have had three 1 h–training sessions and supervision during the first few months of the study. Additionally, adherence to PR was not documented.

Holland and colleagues [30] published a protocol describing a RCT in 166 individuals evaluating the effect of home-base PR using MI compared to outpatient PR. It is an eight-week program and primary outcome will be the immediate change in six-minute walk distance.

Designing the optimal intervention for the promotion of PA in people with COPD is an intensively investigated topic in recent literature [19, 20, 31]. In order to address the recommendations of the ATS and the ERS more accurately, the current study aims to improve long-term self-management and adherence to exercise at home during PR by adding counselling based on MI. This study also aims to gain information about the willingness to actually change habitual behaviour in patients with COPD and evaluates the patients’ perspective on barriers and facilitators towards daily PA. There is no conclusive evidence yet about the best strategy to promote PA and helping people with COPD to change their behaviour. Data from well-designed interventional studies looking at the willingness to actually change habitual behaviour in people with COPD are urgently needed [20]. The results of the study may gain insight into the factors determining physical activity and help leading to novel therapeutic approaches in the care of people with COPD.

### Objective

The overall aim of the present study is to investigate if a PA counselling program during outpatient PR increases PA level in daily life in people with COPD during and after PR.

## Methods

### Design and ethics

**Fig. 1:**
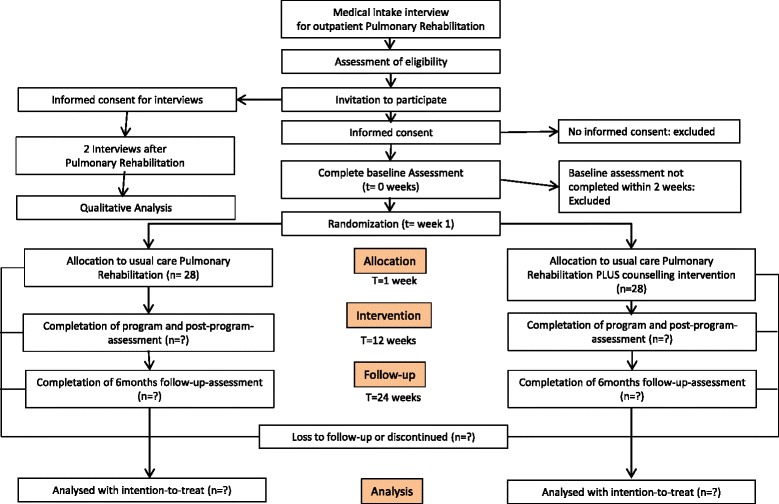
Design and flow of participants through the trial

The study is a prospective two-arm single-blinded randomized controlled trial comparing a PA counselling group (PAcG) with a usual care control group (CG). Figure 1 shows a flow chart of the study. The study was approved by the local ethics committee (Canton Zurich) on 4th May 2015 (*PB_2016–01523*) and inclusion was initiated in October 2015 (still ongoing).

The data collection will be conducted in compliance with Good Clinical Practices protocol and the Declaration of Helsinki principles. The participants with COPD will receive written and oral information about the study. Written informed consent will be obtained prior to baseline measurements. The study is registered on the website of http://www.ClincalTrails.gov with the identifier NCT02455206 as well as the Swiss National Trails Portal SNCTP000001426.

### Setting

This study will be conducted at Canton Hospital Winterthur (KSW; Institute for Physiotherapy and Pulmonary Division), Switzerland. Patients participate in the outpatient PR program “Pneumofit”.

### Participants

Participants fulfilling all of the following inclusion criteria are eligible for the study: Informed consent as documented by signature aged 40–90 years and confirmed COPD (GOLD stages B-D) according to GOLD-guidelines [12], German speaking and planned participation in Pneumofit. The presence of any one of the following exclusion criteria will lead to exclusion of the participant: Mental or physical disability (known mini-mental score < 20) precluding informed consent or compliance with the protocol, patients on morphine medication, primary diagnosis of heart failure, uncontrolled arrhythmias causing symptoms or hemodynamic compromise, severe co-morbidity (acute coronary syndrome, unstable angina terminal renal failure, concomitant pulmonary embolism, very severe pneumonia: CURB65 > 3), severe untreated arterial hypertension at rest (> 200 mmHg systolic, > 120 mmHg diastolic). Criteria for withdrawal are defined as cordial ischemia, fall in systolic pressure > 20 mmHg from the highest value during the test, hypertension (> 250 mmHg systolic; > 120 mmHg diastolic), recurrent exacerbation, interruption of rehabilitation program ≥3 weeks. Withdrawn participants will be contacted 3 month after the last visit. They will be asked if they would like to participate in the follow-up assessment (accelerometer, questionnaires) and focus-group interviews. We will record any adverse events such as injuries, increase of respiratory symptoms or cardiac events as they happen during the outpatient PR.

### Recruitment and randomization

People with confirmed COPD according to the guidelines referred to the pulmonary division of KSW between October 2015 and likely October 2018 will be asked to participate in the study. No public advertisement will be performed. Using a generator (R statistical software) the patients will randomly allocated into blocks of four, within one of two treatment arms. Additionally, stratification is used to ensure good balance of exercise capacity (6MWD). The randomization list will be accessible to the responsible members of the research team. The individual assignment will be kept in closed envelopes, which will be opened after the patient gives his/ her consent to participate in the study. Randomization will be performed by an independent person. For practical reasons participants and therapists are not blinded, but the assessors will be blinded.

### Intervention

#### Pneumofit

Cardio-pulmonary exercise training will be performed according to published guidelines [19, 32–34]. Participants attend the outpatient clinic three times per week (1.5-h sessions) for 12 weeks performing 36 sessions of physical training. The sessions include dynamic strength training for the following muscles: quadriceps femoris, hamstrings, triceps surae, pectoralis major, deltoid, latissimus dorsi and triceps brachi. The dynamic strength training exercises will be performed while seated. Patients start at 70% of their initial one-repetition maximum (1RM) and fulfill 2 cycles of 6-12repetitions of isotonic muscle contractions [32–34] with a resting period of 1–2 min between the series. One repetition maximum (1RM) is the maximum amount of weight one can lift during a single repetition of a given exercise.

When the patients feels able to perform three sets of more than 15 repetitions without any difficulty, effort will be increased stepwise by 5% of the 1RM. Cardio-pulmonary endurance training will be performed on a cycle ergometer or treadmill [32–34]. The initial intensity for cycling will be set at 70–80%Wpeak for 20 min. Increases in workload will be based upon symptom scores. Interval endurance training will be performed according to the recommendations by Gloeckl and colleagues [35]. Participants perform one training per week outdoors participating in a nordic walking class.

#### Experimental group

Participants allocated to PAcG will receive PR plus counselling. PA counselling will be performed by using “motivational interviewing” (MI) techniques. The PA counselling will be provided by two experienced physiotherapists (MSc.), independent to the rehabilitation program, who are profoundly trained and graduated by a 9 days MI course. There are going to be five face-to-face conversations a 30 min between the therapist and one patient during the twelve-week rehabilitation program “Pneumofit” at KSW. Privacy during the sessions will be ensured. The dates of the session will be organised by the receptionists of the physiotherapy institute of KSW and the ZHAW. In case of missing out, the session will be held within the shortest possible timeframe.

##### Evaluation of communication skills of counsellors/fidelity check

If the participant of the PAcG agrees all counselling sessions will be audiotaped. The fidelity check evaluates the general session content, as well as random 20-min segments of the conversations which will be assessed by an external independent MI-expert according to the Motivational Interviewing Treatment Integrity (MITI 4) [36] criteria. Feedback will be provided to the counsellors to strengthen their skills and consistency with the study intervention [37].

#### Controls

Participants allocated to the control group will receive usual care PR.

### Measurements

Before beginning of PR program, baseline pulmonary function test and other routine assessments will be performed. In addition, study specific assessments will be conducted. The patients are asked to wear an accelerometer during seven consecutive days. Furthermore, the patients are invited to fill out four different questionnaires (*BREQ-3, SCPA, IPAQ*, and *CRQ*). Assessments will be conducted at baseline (week 0), after 3 month when finishing PR (week 12), and patients will be contacted for the follow-up assessment (week 24). Additionally, participants will be asked to take part in interviews for a qualitative analysis.

#### Daily physical activity (PA)

PA will be measured by a multisensor accelerometer *(SenseWear Pro® armband; BodyMedia, Inc., Pittsburgh, PA, USA),* which is worn on the upper right arm. The device estimates energy expenditure (EE) using measurements from a biaxial accelerometer and sensors that quantify galvanic skin response, heat flux and skin temperature. The biaxial accelerometer records the number of steps per day and the duration of PA [38]. The number of steps per day, metabolic equivalent (MET), total energy expenditure (TEE), and the physical activity level (PAL) will be used in the present study. Primary outcome is number of steps per day. MET, TEE, PAL are secondary outcomes. PAL is calculated using TEE and sleep expenditure as a surrogate for resting energy expenditure (REE) (PAL = TEE/REE). The patients will be instructed to wear the accelerometer continuously during seven consecutive days, except when bathing or showering. The *SenseWear Pro®* armband was validated to accurately measure PA and quantify EE in patients with COPD [28, 29, 39].

#### Behaviour exercise regulation questionnaire (BREQ-3)

The BREQ-3 [40] considers the individuals’ motivation towards exercise. The questionnaire comprises 24 items relating to five motivation types from the Self-determination Theory [41]. Each item is measured on a scale, from 0 (“Not true to me”) to 4 (“very true to me”). The BREQ-2 was already used in the context of PA research [42, 43]. BREQ-3 was translated into German following guidelines for translation and cross-cultural adaption of self-report measures [44–46].

#### Physical activity stage of change assessment tool (PASC)

The Physical Activity Stage of Change Assessment Tool [47] was designed to assess the patients’ willingness to change their habitual behaviour using a brief series of four questions based on the transtheoretical model [38]. PASC was translated into German following guidelines for translation and cross-cultural adaption of self-report measures [44, 45, 48].

#### Awareness of physical activity

The *awareness* of the PA level will be evaluated by the German International Physical Activity Questionnaire (IPAQ) [49, 50]. The IPAQ considers a 7-day recall period, identifying PA undertaken in the morning, afternoon and evening. IPAQ quantifies PA by using the time send on walking, moderate and vigorous PA. The IPAQ was used in patients with COPD in previous research [51].

#### Compliance to PR

Compliance is defined as the number of sessions completed divided by the total number of sessions prescribed and will be expressed as a percentage. The Program Pneumofit at KSW consists of minimal 24 and maximal 36 training sessions.

#### Health-related quality of life (CRQ)

The Chronic Respiratory Disease Questionnaire (CRQ) is a disease-specific assessment for evaluating COPD [52]. The CRQ-Questionnaire has been validated to quantify the impact of disease on daily life and well-being [53]. The CRQ consists of 20 items, which can be divided into four domains: dyspnoea (five items), fatigue (four items), emotional function (seven items) and mastery (four items). The patients will be asked to rate each item on a 7-point scale from 1 (maximum impairment) to 7 (no impairment). Higher scores imply better self-reported disease-specific QOL. The total CRQ score, calculated as the mean of the scores on the four dimensions of the CRQ, will be used for further analysis.

#### COPD assessment test (CAT)

The CAT score is a unidimensional patient-completed questionnaire assessing globally the impact of COPD (cough, sputum, dysnea, chest tighteness) on health status [54]. The CAT scores range from 0 to 40. Higher scores denote a more severe impact of COPD on a patient’s life. The difference between stable and exacerbation patients was five units. No target score represents the best achievable outcome.

#### Cardiopulmonary exercise capacity

The incremental work-rate test permits the evaluation of both submaximal and peak exercise responses, providing several indices relevant to the evaluation of patients with respiratory diseases. Use of the IET can also rule out certain medical conditions that pose a risk for exercise interventions, thus increasing their safety. Also, exercise tests are reliable and consistently responsive to rehabilitative and pharmacological interventions. Symptom-limited cardiopulmonary exercise testing will be performed using an electronically braked cycle ergometer (Ergoselect 200). After an initial 3 min of unloaded pedalling, the work load will be increased automatically by 5–8 watts every minute until the patient could no longer continue the required cadence of 55–65 rpm due to dyspnea or exhaustion.

Maximum work rate (Wmax) will be defined as the highest work level that was reached. Oxygen saturation (measured via pulse oximetry) and heart rate (measured via electrocardiography) will be recorded continuously throughout exercise and during recovery.

#### Six-minute walk test (6MWT)

The Six-minute walk test (6MWT) will be performed according to the guidelines published by the ATS [55] with a standardized encouragement in a 30 m corridor. An experienced technician will supervise it. Before starting the 6MWT, patients will be seated for three to 5 min. The Spirometry will be performed prior to the test. The 6MWT will be followed by a recovery phase, where the patient will rest on a chair. Total walking distance, Borg’s dyspnoea score and the subjective limiting factor (according to the patients’ interpretation) will be measured.

#### Sit to stand (STS)

Furthermore, the sit-to-stand-test (STS) will be performed according to Puhan and colleagues [56]. Using the STS test protocol, an experienced physiotherapist will ask patients to sit down on a chair (height 46–48 cm) without arm rests, keep their legs apart with about 90 degrees knee flexion and aligned with their hips, and to hold their hands stationary on their hips. Patients will be asked to stand up and to sit down once or twice in order to familiarize them with the task and to assess its feasibility and safety. A physiotherapist instructs patients about the duration of the test (1 min) and to perform as many repetitions as possible allowing for short breaks if needed but without using the arms for support. The physiotherapist starts the test by giving the command “attention, ready, go”. When 15 s are left patients will be told “You have 15 seconds left until the test is over”.

#### Pulmonary function

Participants will receive spirometry, whole body plethysmography and diffusion capacity measurements according to the ATS/ERS guidelines with a commercially available system (Body 500™, ZAN, Oberthulba, Germany) [57]. Post bronchodilator spirometry will be performed after inhalation of 4 × 100 μg salbutamol**.** Maximal inspiratory mouth occlusion pressure after 100 ms (PO.1max) will be measured as previously described [57].

#### Barriers and enablers PA

Individual, semi-structured interviews will be performed in a subgroup of 16 participants in order to gain more detailed information about “barriers and enablers” of participation in daily-life activities. Participants will be invited to two interviews, one right after PR the other after 3 months follow up. Discussions will be audiotaped, transcribed at verbatim, coded and categorized according to upcoming themes. A content analysis will be performed.

Research questions are:

- What are motivating and limitating factors to be physically active for patients with COPD?
- Does PR change the patients’ attitude towards PA? How?
- What can we learn for “Pneumofit”?

### Statistics

#### Statistical model

Applying random intercept models, we model the continuous response *y*_*gtk*_ (number of steps) of subject *k*(*k* = 1*, . . ., n*) at time *t* (baseline *t* = 0, follow-up *t* = 1) in treatment arm *g* (control: *g* = 0, intervention *g* = 1). The model is

*y*_*gtk*_ = *μ* + *γ*_*g*_ + *τ*_*t*_ + (*γτ*)_*gt*_ + *S*_*k*_ + (*Sτ*)_*k* , *t*_ .

Fixed effects: *μ* is the mean outcome in the control at baseline, γ1 is the difference at baseline between the mean of the intervention and control group (γ0 = 0) - in randomized controlled trials, we expect that *γ*_1_ = 0, *τ*_1_ is the change from baseline to follow-up of the control means (*τ*_0_ = 0), *δ* = (γτ)_11_ is the difference in change from baseline between intervention and control means (for *g* ≠ 1 or *t* ≠ 1: (γτ)*gt* = 0). This is the quantity of interest!

Random effects: the random effects *S*_*k*_ *,* (*sτ*)_*k,t*_ are normally distributed with mean 0 and variance ${\sigma}_s^2$ *,* ${\sigma}_{s\tau}^2$, respectively. Thus, the (unconditional) variance of the response *γ*_*gtk*_ is ${\sigma}^2={\sigma}_s^2+{\sigma}_{s\tau}^2$. The *sk,* (*sτ*_*k* , *t*_)_*k,t*_ describe the variation of subjects in time-invariant and time-varying part. Subject autocorrelation The subject autocorrelation defined by${\rho}_s=\frac{\sigma_s^2}{\sigma_s^2+{\sigma}_{s\tau}^2}$describes the correlation of the subject specific scores *s*_*k*_ + (*sτ*)_*k* , *t*_.

#### Sample size calculation

We make the assumption based on prior literature findings [27] that our counselling will yield a larger difference between the two groups: after 3 months of therapy, the patients in the experimental group walk 3000 steps per day (SD = 4000) more than the patients in the control group.

The randomized trial will be analysed using ANCOVA with the outcome at baseline as a covariate. The approach to power calculations can be resumed: a trial of (1 –*ρ* ^2^)*n* subjects analysed via ANCOVA has the same power as a trial of *n* subjects analysed on the follow-up scores, *ρ* representing the subject autocorrelation between baseline and follow-up. The design effect is thus given by (1 − *ρ*2). For an equality study, we have the hypothesis *H*_0_: *δ* = 0*, H*_1_: *δ* ≠ 0. With an effect size of 3000/4000 = 0*.*75, the per-group sample size is, using a power of 0.8, *α* = 0*.*05 and a conservative estimation of the within-subject autocorrelation of ρ = 0*.*5, *n* = 22. Considering a drop-out rate of 25%, a total of 56 patients (28 in each group) need to be recruited.

#### Statistical analysis

The data will be analysed with ANCOVA using baseline values as covariates. As described above, this analysis is the most efficient. In the analysis stage, no null-hypothesis significance testing-procedures (NHSP) will be used. Instead, we will use a modern Bayesian approach with Markov Chain Monte Carlo methods (MCMC) and non-informative priors on the unknown quantities. Bayesian inference is the reallocation of credibility across the space of candidate possibilities for the unknown quantities and gives much richer information than classical frequentist procedures. The Bayesian approach results in the posterior distributions of all unknown quantities (parameters and missing values), therefore allowing unambiguous probability statements about the quantities of interest those are not possible in a frequentist framework. In addition, the computation of derived quantities (such as contrasts of interest) is straight forward. Ninety-five percent Bayesian credible intervals will be constructed for the parameters of interest. All analyses will be performed using the R statistical software R version 3.3.2 (2016–10-31) [58] and JAGS (Just Another Gibbs Sampler) [59]. JAGS is designed to work with the R language. From within R, we will use the rjags-package [60].

## Discussion

The development and implementation of successful strategies to improve PA are in the focus of both, medical research and public health [8–11]. The minimal important difference regarding a proxy of PA has not been defined yet in people with COPD. PA recommendations for the elderly state that 30 min of moderate intense physical activity on at least 5 days per week in order to improve or maintain their health status [61]. But even these recommendations are not realistic for people with very severe COPD. There seems to be a common agreement that PA promotion in people with COPD has to incorporate the patients’ personal values and goals, symptoms, and motivation status [20, 24, 25, 62, 63]. Donaire-Gonzales and colleagues [64] evaluated the interaction of quantity and intensity of PA in their effects on COPD hospitalisation risk. For people with low average PA intensity the risk for COPD hospitalisation was reduced by 20% for every additional 1000 daily steps. Contrary and a little surprising, adding steps to an anyway high-intensity PA does not produce any risk reduction. Reducing sedentary behaviour is especially beneficial for people with low PA. Currently, it is still on debate which strategy might be the best to improve PA in people with COPD. Recent literature shows a tendency to patient-centred interventions like counselling [19]. This study aims to investigate if a PA counselling program based on the principles of MI, during PR increases PA level in daily life in people with COPD. Using a mixed-method-approach will gain multifaceted knowledge about barriers and facilitators about the acceptance and effect of this strategy.

It might be a disadvantage not to offer direct feedback like a pedometer to every participant [19]. However, if a participant has the idea to use a step counter (pedometer, mobile phone, etc.) as part of his strategy to be more active, we support this purpose.

The accelerometer SenseWear is especially accurate and validated in this patient group [39, 65], but not easy accessible in clinical practice. The device is much more uncomfortable and unattractive than other devices like a smart watch. Furthermore, the SenseWear device production was stopped in 2015. Anyhow, we decided to stick with the SenseWear.

If this strategy is approved to be effective PA counselling might be implemented as an integral part of PR Pneumofit.

## Abbreviations

6MWD: Six-minute-walk-distance
ANCOVA: Analysis of covariance
ATS: American Thoracic Society
BREQ: Behaviour exercise regulation questionnaire
CAT: COPD Assessment test
CG: Control group
COPD: Chronic obstructive pulmonary disease
CRQ: Chronic respiratory questionnaire
ETS: European Respiratory Society
IPAQ: International Physical Activity Questionnaire
KSW: Kantonspital Winterthur
MCMC: Markov chain monte carlo methods
MET: Metabolic equivalent
NHSP: Null-hypothesis significance testing-procedures
PA: Physical activity
PACG: Physical activity counselling group
PAL: Physical activity level
PASC: Physical activity stage of change assessment tool
PR: Pulmonary rehabilitation
REE: Resting energy expenditure
RM: Repetition movement
STS: Sit-to-stand
TEE: Total energy expenditure
